# Analysis of Burnout and Psychosocial Factors in Grassroot Football Referees

**DOI:** 10.3390/ijerph18031111

**Published:** 2021-01-27

**Authors:** Natalia Orviz-Martínez, María Botey-Fullat, Sergio Arce-García

**Affiliations:** 1 Escuela Superior de Ingeniería y Tecnología, UNIR-Universidad Internacional de La Rioja, Avenue de la Paz, 137, 26006 Logroño, Spain; sergio.arce@unir.net; 2 ESIC-Business & Marketing School ESIC, Avda. de Valdenigrales s/n, 28223 Madrid, Spain; maria.botey@esic.edu

**Keywords:** burnout, referee, soccer, violence, soccer fans

## Abstract

The aim of this paper is to analyze the interrelationships between the burnout and different psychosocial variables to which the grassroots football referee is exposed, in particular, associated with the influence of the environment and the level of verbal and physical aggression. To this end, a questionnaire was developed, consisting of items from *the Maslach Burnout Inventory-General Survey* *(MBI-GS)* and various self-constructed items designed to find out these psychosocial variables. First, a study of the structure of the form was carried out. Second, a structural equation model was designed in order to test the causal relationship between the variables under consideration. The results obtained point to the validity of the proposed theoretical model. It is recommended to initiate training programs for this group aimed at strengthening personal coping and social support strategies, which can help minimize the evolution of this syndrome.

## 1. Introduction

Football is the most popular competitive sport in Spain and much of the world, which produces more economic income and generates more fans from both its highest level (elite professional sport) to grassroots football. Its ultimate judge is the referee, a key figure who oversees ensuring that the rules of the game necessary to compete are observed, that players respect the authority and play fairly and to transmit the values of the sport [1]. Therefore, the referee’s role is to provide a safe environment for the sporting activity and to maintain the integrity of the sport [2].

In addition, soccer, as a sport of great popular preference and constant media coverage [3], presents a large public exposure, where the media promotes its possible prospective mistakes. All of this can cause and/or intensify feelings of discomfort, stress and disability in referees [4,5]. Therefore, on many occasions, referees can even be perceived by many people as corrupt and prone to act with dubious intentions [1].

The main motivations that influence refereeing are related to intrinsic aspects such as passion and love for sport, achieving certain goals in refereeing (experience to be able to referee at a higher league level), obtaining satisfaction, social relationships, travel, fun, the feeling of challenge and competition [6,7,8,9]. In contrast, extrinsic reasons such as economic retribution are much less relevant [10,11]. Furthermore, the degree of support offered by the organization is an important factor in the referee’s commitment to continue refereeing [12].

The initiation of arbitration in grassroots football usually occurs in a solitary manner (except first division of juveniles and amateurs’ categories, there are not assistant referees, only one referee), which adds to lack of experience, support and psychological resources, foregoing activities of personal and professional nature, and the suffering of certain types of violence. The above factors make that, even if it is the collective that needs the most support, it is one of the least attended [13,14]. In this context it is common for any referee, initially oriented to intrinsic goals related to sports enjoyment, relationship with people and personal development, to progressively lose his motivation and experience higher levels of stress, which is associated with the risk of suffering the so-called burnout or burnout syndrome and the resignation to arbitration [15,16,17,18].

Referees may suffer burnout throughout their arbitration career. Their work during the course of the match presents various difficulties, as they must apply rules in short intervals of time and under the pressure of players, coaches and spectators. If these practices cause them insecurity, they can increase the lack of motivation and loss of enjoyment [19], increasing the chances of making mistakes and, consequently, receiving negative criticisms [20,21]. In this situation, the emergence of excessive tension and anxiety is enhanced in the referees, due to internal and external demand not to fail [22] and is therefore a group capable of burnout.

## 2. Theorical Background and Hypothesis

Burnout can be defined as a prolonged response to chronic stress, which causes fatigue and emotional exhaustion or loss of physical and mental energy, due to problems in interpersonal relationships, especially at work [23], which can manifest itself, not only in the workplace, but also in other contexts, such as the sport. In this sense, different studies carried out on burnout in the field of soccer have focused mainly on coaches and players [24,25,26,27,28], although, logically, this phenomenon also affects referees.

The referee is a key figure in promoting a quality sport experience [12], but on numerous occasions, before, during and after matches, he is the center of controversy, physical attacks [29] and psychological attacks by players, technical and public bodies [30], which are usually news in different media [31].

In [32] a review of published studies up until March 2015 on burnout in the sport was conducted, in which the scarcity of work examining this group was proven. To this problem of a lack of research, we must add the particular case of the grassroots football referee [33], numerous and out of the media spotlight, since the few studies, but with increasing attention, focus mainly on the case of the major professional leagues [34,35,36]. In this area, a burnout prevalence rate of 2.44% was identified among the collective, with a higher trend among the main referees of the Spanish First Division and with more experience, also finding some relationship between burnout, stress, anxiety and social support, already observed in previous works [18,37,38].

Under this frame of reference, the objective of this study is to identify and empirically prove whether the environment and violence influence burnout in a sample of grassroots football referees.

For this research, five factors are identified, three of them—emotional exhaustion, cynicism and professional effectiveness—from Maslach’s questionnaire, and two more—influence of the environment and verbal and physical aggression—that are incorporated into this research. These variables were added in accordance to a study done together with experts, indicated in the methodology, in order to determine and empirically prove whether the environment and physical or verbal aggression influence professional effectiveness, as reflected in the list of internal and external stressful situations for the performance of the arbitration of the collective (LISEA) initially developed in basketball referees [39], and later applied in soccer referees [40]. All of this would lead to a decrease in performance efficiency, as it was demonstrated [41].

Precisely one of the psychological factors that affect and influence their decisions is physical and emotional exhaustion [41], which can lead them to make judgmental mistakes during the game, which can have an impact on professional inefficiency [1]. Due to the exposed scenario, it is possible to postulate the following hypothesis:

**Hypothesis** **1** **(H1):**  *Emotional exhaustion causes professional inefficiency.*

On the other hand, emotional exhaustion causes in soccer referees a sense of reduced realization and devaluation of the sport [23]. Referees also must deal with interpersonal conflicts arising from criticism by players, coaches and spectators [20,21]. They are often verbally attacked, sometimes even physically [17], establishing a fear-dominated environment that can generate emotional exhaustion.

Previous research determine that referees are emotionally sensitive to the influence of the environment and psychological aggressions by viewers, leading them to problems of stress, attention, exhaustion and lack of concentration while performing their tasks [42]. In an investigation carried out in football referees in Brazil, a significant relationship of burnout levels with respect to the zones or areas where they were refereed, due to the social and organizational context of some neighborhoods versus others [43]. In this line, [44] they found that they identify especially the viewers, and to a lesser extent players and coaches as sources of attacks or abuses towards them, mainly verbal in type [45]. All of these behaviors exert, together, unwanted pressure that can cause psychological problems when making decisions during the game [46], generating exhaustion and loss of interest in work. In fact, research on referees in other sports; indicate that the number of spectators and protests that referees may receive is directly related to burnout levels [47]. With this same approach it is established that:

**Hypothesis** **2** **(H2):**  *The influence of environment and the level of physical and verbal aggression suffered by referees increases emotional exhaustion and cynicism.*

The existence of doubts, about the belief of one’s ability to perform the job or perceived effectiveness, produces, in the long run, a decrease in performance and generates stress [48] and insecurities about the usefulness of their work, that is fed back and increased over time. These results support the existence of “negative spirals” [49] which the presence of obstacles creates a lack of professional effectiveness, which in turn triggers the burnout process [50]. In this way, when judged incapable of fulfilling their role, a perception of inadequacy to work appears in the referee [51]. These considerations lead to pose the following hypothesis:

**Hypothesis** **3** **(H3):**  *Professional inefficiency generates lack of interest in work (cynicism).*

## 3. Materials and Methods

### 3.1. Sample

The collaboration of different Spanish soccer federations was solicited, accepting their participation the Technical Committee of Referees of the Asturian Football Federation. In order to obtain the data, an ad hoc questionnaire was prepared, which sample was administered online through computers or mobile devices anonymously, sent by said Committee.

At the time of the study, in mid-2019 the number of referees ascribed to the committee was 415, of which 391 (94%) were men and 24 (6%) women. Due to the climatology and orographic conditions of Asturias, outdoors soccer is only held with players ten years old and older, while those under that age play futsal in sport centers. Therefore, the grassroots football collective in Asturias falls into categories according to the age of the players: youth (10–11 years), children (12–13), cadet (14–15), juvenile (16–18), and amateur soccer (above 18 years, in regional categories up to Third Division).

The questionnaire was administered to the total population (415 referees of the Asturian Football Federation) and was voluntarily completed by 203 (49%) referees of the total collective. The sample was obtained incidentally or non-probabilistic.

The characteristics of the sample are shown in Table 1. Most of the grassroots football referees are men (94%) The ages of the sample referees range from 14 to 41 years. The average age of the sample is 23.5 years. On the other hand, most of them regularly arbitrate in 3 to 5 different categories at the same time. No one of these referees are professionals, but they have a little paid per match depending on the category arbitrated.

### 3.2. Instruments

An ad hoc data collection form was constructed, with a series of questions for identifying users by referee type and their previous experiences regarding environmental aspects and the MBI-GS questionnaire [52] applied to non-caring professions. This methodology was adapted and validated for Spain according to the Technical Note of Prevention number 738 of the National Institute of Safety and Health at Work, made by [53]. Therefore, this question program consists of four distinct blocks:

- Block 1: sex, age and arbitration categories (youth, children, cadets, amateur and adults).
- Block 2: Three items that reflect a dimension related to different environmental aspects that can condition the work of referees in matches: Does the name of the teams affect you in your arbitration? (previous 1); Are you affected by the existence of certain known players? (previous 2); and are you affected by the presence of a media or recording medium? (previous 3).
- Block 3: Three items referring to a possible dimension associated with the level of verbal and physical aggressions suffered by referees: Have you ever been disrespected by any coach? (previous 4); Have you ever been disrespected by the public? (previous 5); Have you ever experienced physical violence? (previous 6). For the study of the items used in blocks 2 and 3, the validity of content was quantified by judge’s discretion with the Aiken’s V coefficient (V), which allows quantifying the relevance of the items with respect to a content domain from the valuations of N judges [54]. To do this, a group of eight experts in psychology and soccer arbitration, completed a test assessing two questions for each of the items (1.-relevance of the area, 2.-quality of the writing) valued with Likert scale of 5 (TD, D, N, A, TA). From this valuation, the Aiken V index was obtained for each item obtaining a value greater than 0.91 for all items in each question. Finally, in block 2 index V was 0.92 and 0.91 for questions 1 and 2 and in block 3 of 0.93 and 0.94, which makes it possible to consider these two blocks valid for the content domain representing [55].
- Block 4: 16 items extracted from the MBI-GS questionnaire applied to non-caring professions [56]. As is known, this instrument evaluates the three dimensions that characterize this syndrome: emotional exhaustion, characterized by mental wear and tear, the feeling of not being able to give more of themselves emotionally and an absence of energy and enthusiasm; cynicism, associated with the development of negative attitudes, such as insensitivity and cynicism towards others; and professional inefficiency, determined by low self-esteem and the inability to withstand pressure and be satisfied with the results obtained. High scores in emotional exhaustion and cynicism and low scores in professional efficacy are indicators of burnout.

Regarding the last block of the questionnaire, an adaptation of the MBI-GS items was made to the characteristics of the activity of the grassroot football referee collective, obtaining the questions shown in Table 2. This adaptation was developed by a group of independent experts from different Spaniard public and private universities, who made different suggestions and recommendations that were considered in the final version of the instrument.

There are a large figure number of studies conducted based on the application of the MBI inventory that support the three-dimensional proposal of the syndrome, however, in various types of populations it has an unstable factorial validity [57]. Some research suggests modifications, diverse ways of interpreting items and in other cases the removal of some of them [58,59,60,61].

One of the items of the cynicism dimension in the original questionnaire “I want to simply do my job and not be disturbed” was removed from the analyses due to the problems encountered in previous investigations into the construct validity of that instrument [62]. Also, some deficiencies were detected in other items in terms of their understanding and interpretation (two of professional efficiency and another of cynicism), yet they were initially maintained in the model.

The response scale used in all items was the original one of type Likert, with seven possible values (from 0, never, to 6, every day). The same scale of answers was also applied in the previous questions that are part of the rest of the questionnaire.

### 3.3. Techniques for the Processing of Information

For the analysis of the internal structure of the questionnaire used, different data analysis procedures were carried out.

First, a descriptive analysis of the different variables involved in the study is carried out, calculating the mean and standard deviation of each group of grassroots football referees in the sample, such as representative descriptive statistics.

Secondly, two important characteristics for any measurement of an instrument or questionnaire in the social sciences were studied: reliability (consistency or stability of a measurement) and validity (measures the degree to which the test presents the structure of factors or dummy variables for which it has been defined). These two qualities are key to the testing of the “psychometric strength” of the instrument [63].

Reliability was obtained through the coefficient ω (omega) for each of the constructs, thus obtaining an indicator of internal consistency.

For study of the validity of the factorial structure of the MBI-GS instrument (block 4), following the classic recommendation [64], an exploratory factorial analysis (EFA) and a confirmatory factorial analysis (CFA) were applied, with a complementarity approach, checking previously the correlations between factors. The CFA provided information on how to solve potential problems in the adjustment of the EFA that begins with the known factorial structure proposed by the MBI-GS instrument.

Finally, to study the relationships between the variables treated and their influence on the factors associated with burnout syndrome, a structural equation model (SEM) was carried out using the sum of the final five variables of three blocks of the created questionnaire, as observed variables.

SEM models are based on linear regressions. Although the regression analysis has to do with the dependence of a variable on other variables, this does not necessarily imply causality. To indicate causality, a priori or theoretical considerations must be taken into account. However, the scientific community considers SEM to be the most powerful and appropriate methodology for analyzing the plausibility of a causal relationship [65].

SEM model is a multivariate statistical tool for testing and estimating causal relationships from statistical data and informed hypotheses about causality. Among the advantages of using this methodology are the simultaneous testing of the direct relationship, the indirect and total relationship between variables, the inclusion of more than one dependent variable and their respective measurement errors. Among the limitations, it is mentioned that although it works well with relatively small samples, a sample size of no less than 200 observations is recommended to potentiate the results of the model [66]. This methodology was considered appropriate for this investigation, since it provides an empirical verification of the causal relationships between the variables that have been tested in previous studies. All analyses were performed with the AMOS 23.0 program (IBM, Chicago, IL, USA) and SPSS 24 (IBM, Chicago, IL, USA).

## 4. Results

### 4.1. Descriptive Amalysis

To perform the descriptive analysis, the main descriptive statistics (mean and standard deviation) of each factor of the sample were calculated. Specifically, three analyzes have been carried out based on gender, age and the category refereed.

The proportion of women in the sample is very low (4.4 %), although the difference between one sex and the other in terms of emotional exhaustion and cynicism is noteworthy (Table 3). Women show greater emotional exhaustion and perceive less efficiency in refereeing. It should be noted that of the 9 women in the sample, 7 referees in the higher category, but none of them arbitrate in this category exclusively.

The descriptive age analysis is carried out by grouping the ages into different intervals, so that the number of arbitrators who make up each group is homogeneous (between 30 and 37 referees).

The age range 23–24 has the highest average values for each factor, except for the effectiveness sense, in which the youngest age group stands out, possibly because of its lack of experience. Standard deviations are similar for each age group within the same factor (Table 4).

Regarding the values obtained by grouping the sample by the categories judged, the amateur category has the highest values in all factors except emotional exhaustion, which has a higher average for the group of referees it referees in the cadet category. That is, referees who arbitrate in the amateur category perceive greater influence of the environment and greater aggressiveness. In addition, they also have high values in the factors associated with MBI-GS. However, it is the youth category that has the lowest values in all factors. For standard deviations, similar values are observed between groups of grassroots football referees for the same factor (Table 5).

### 4.2. Reliability and Validity of Each of the Blocks Studied

The reliability of the dimensionality of each of the blocks studied has been verified by calculating the omega coefficient (ω coefficient). The ω coefficient obtained takes values greater than 0.7 for each of the items belonging to each dimension considered, and therefore, it is confirmed the reliability of each factor studied in this analysis [67].

Items included in the environment factor show moderately high correlations with the total of their factor (between 0.44 and 0.53).

In the dimension level of verbal and physical aggression the correlations are high, except for the item “Have you ever experienced physical violence?” (Previous 6), whose correlation 0.23. The items contained in the emotional exhaustion factor have the highest correlations with the total of their dimension (between 0.66 and 0.83). In the case of the first two items of the cynicism dimension, the correlations are also considerable (0.66 and 0.60, respectively). However, the other two items, C3 and C4, have a low correlation with the total of their scale (0.29 and 0.41, respectively). Finally, the items of the professional efficiency dimension have the most homogeneous correlations compared to the rest of the dimensions, being moderately high (between 0.42 and 0.54).

As can be seen in Table 6, which calculates Pearson’s correlation coefficient R to analyze the relationship between the different factors, most correlations are moderate (between −0.47 and 0.25), except for that found between the dimensions of emotional exhaustion and cynicism, whose value is 0.61 (*p* < 0.01). In this regard, consideration is given to the possibility that the items of the latter factor (cynicism) have not been interpreted correctly by the participants due to the different meanings of the word cynicism. Finally, it is emphasized that some of the correlations have a negative sign, in particular those related to professional efficiency, which is in line with the conceptualizations on the MBI-GS instrument, which highlight the opposite relationship between the dimensions of exhaustion and cynicism with professional effectiveness.

The validation of the dimensionality of each of the blocks studied has been made through exploratory factor analysis (EFA). This is intended to verify whether the questionnaire used shows a relatively consistent internal structure. However, as is known, this type of analysis is not a sufficient evidence to study the dimensionality of an instrument, even if it is an important first step, since factorial solutions are indeterminate and therefore do not, in themselves, constitute mathematical evidence of the correspondence between the data and the underlying structure [68]. Therefore, a confirmatory factorial analysis (CFA) has also been carried out, with the purpose of proving the extent to which the hypothesized model is compatible with empirical data, allowing us to obtain a measure of the goodness of the fit to the data.

#### 4.2.1. Exploratory Factor Analysis (EFA)

First, an exploratory study of the factorial structure of different instruments involved in the model, associated with block 2 (environment influence), block 3 (verbal or physical aggression) and block 4 (MBI-GS questionnaire with the factors emotional exhaustion, cynicism and professional effectiveness), were studied separately.

On one hand, the validity of the MBI-GS instrument (block 4) was studied with a factorial analysis with the varimax method of maximum likelihood and orthogonal rotation. Previously, two tests were performed: the Kaiser-Mayer-Olkin coefficient (KMO) and the Bartlett sphericity test, the value obtained for KMO was 0.83, which shows an underlying structure of relationships between variables. Bartlett’s sphericity test values (approximate chi-squared = 1438.23, gl = 105, *p* = 0.00 < 0.05) indicate that there is a significant relationship between the items and the constructs or factors considered (Table 7).

Some items showed presence in two factors (EE5, EF4). In general, the factors obtained with EFA largely coincided with the constructs proposed by the Maslach Burnout instrument, except for the cynicism dimension, which unfolded into two factors, each consisting of two items, with C3 being the one with the lowest coefficient. In addition, within the effectiveness factor, EF2 shows a very low coefficient in the professional efficacy factor (0.487).

As for block 2 (environment influence) a single factor with self-value greater than 1 was obtained that summarizes a 42.2% of the total variance, the KMO = 0.65 and Bartlett’s sphericity test (approximate Chi-squared = 95.9, gl = 3, *p* = 0.00 < 0.05) indicate that there is a significant relationship between the items and the construct under consideration.

For block 3 (verbal or physical aggression) a single factor with self-value greater than 1 was obtained that summarizes 40% of the total variance, KMO = 0.56 and Bartlett’s sphericity test (approximate Chi-squared = 80.9, gl = 3, *p* = 0.00 < 0.05) indicate that there is a significant relationship between the items and the construct under consideration.

#### 4.2.2. Confirmatory Factorial Analysis (CFA), Block4 (MBI-GS Items)

**Figure 1:**
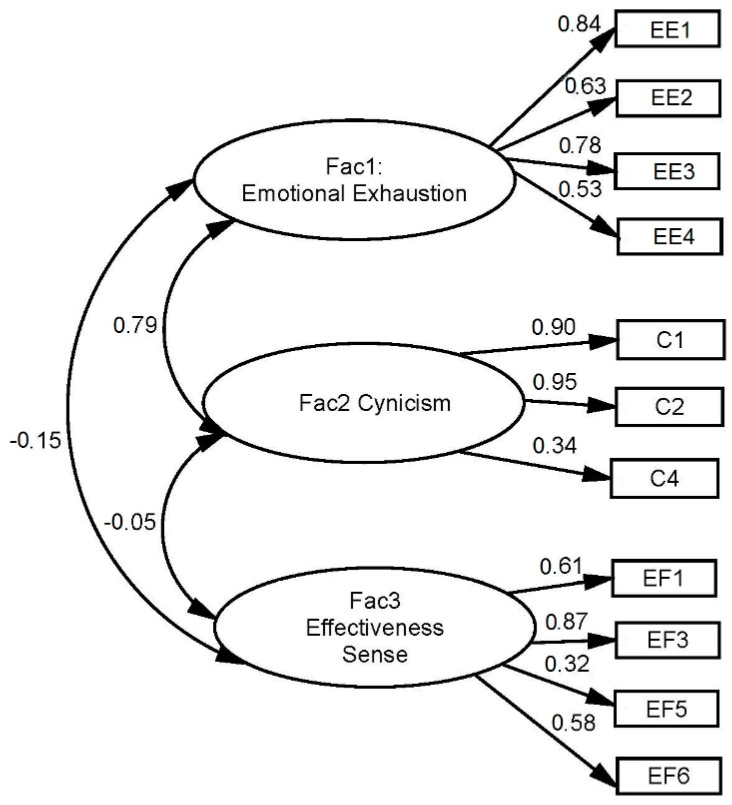
Confirmatory factor analysis of the MBI-GS instrument composed of the factors defined by the instrument and their covariance. The goodness of fit results allows confirmation of the structural validity of the instrument in the sample. Note: EE1, EE2, EE4; C1, C2, C4; EF1, EF3, EF5, EF6 respectively represent the items of the emotional exhaustion factor, cynicism factor, and effectiveness sense factor, of the MBI-GS instrument.

Based on the structure proposed by the MBI-GS instrument, a model was defined with three constructs (emotional exhaustion, cynicism and professional efficiency) with the method of maximum likelihood. Taking into account the indications of [69], the results of the EFA and the theoretical knowledge of the items that make up the MBI-GS in the soccer arbitration environment, the final model with indicators of good fit was the one shown in Figure 1, composed of eleven items (items EE5, C3, EF2 and EF4 were deleted). The removal of these items already identified in the EFA as unsuitable, provided in the CFA a substantial improvement of the model, adapting the chi-square and other adjustment indicators. Numerous studies analyze the possible elimination of some items by adapting the MBI-GS to different languages [70,71,72,73] or to special environments, such as football arbitration [56].

The measurement model presented in Figure 1 was compared, in terms of statistical adjustment, with a model that does not include correlations between the residues of the indicators of the emotional exhaustion construct, so that, the inclusion of correlations transitions the model from an acceptable model to a well-adjusted model.

These correlations reflect in the model the relationship between sources of variability of the associated items. The theoretical basis that leads to include correlations between residues in the analysis is that items EE1, EE2, EE3 and EE4 have great similarities, all present a part related to another item that is not present in all at once, so it is not integrated into the Emotional exhaustion construct. For example, items EE2 and EE4 present in common the stress of work and its exhaustion effect without reflecting in this common part the themes associated with emotions. Similarly, there is a relationship between EE2, EE3 and EE4 that reflects the tiredness of prolonged stress throughout the working time.

The model obtained presents all significant estimators at 95% probability, being item EE1 the one with the highest standardized weight on the emotional exhaustion factor, EF3 has the greatest weight over the professional efficiency factor and C1 the one with the greatest weight in the cynicism factor. The covariance between cynicism and professional effectiveness is low and not significant, however, it has been maintained in the model respecting the MBI-GS model since the goodness estimators of the model remain within the ranges of good fit; furthermore, the negative sign of this covariance (−0.05) is of interest, reflecting the opposite direction of these two concepts (the greater the cynicism, the less professional efficiency).

The Chi-square value is 5.210 with 37 degrees of freedom (*p* = 0.052). However, these statistics are not usually used as good indicators of the goodness of a model, because their fluctuability depends largely on the sample size, with small samples usually getting better adjustments [74]. Therefore, other indicators were valued, such as the comparative fit index (CFI) [75] and the root mean square error of approximation (RMSEA) [76]. All indicators are in the good adjustment interval (Table 8). These indicators show that there are no discrepancies between the theoretical model proposed and the empirical data, i.e., that the model of our questionnaire is consistent.

This model shows the existence of significant covariance between the constructs of the MBI-GS instrument, with the constructs emotional exhaustion and cynicism being the most correlate (β = 0.79), in addition, the covariance between emotional exhaustion and professional effectiveness has a negative coefficient (β = −0.15), as would be expected according to the theoretical model of the MBI-GS instrument. As for the correlation between cynicism and professional efficacy is low (β = −0.05), with the expected sign but not significant (*p* = 0.50), however, the information it provides is maintained in the model as of interest and because, nevertheless, the model maintains acceptable levels of fit.

### 4.3. Relationships between Factors Studied

**Figure 2:**
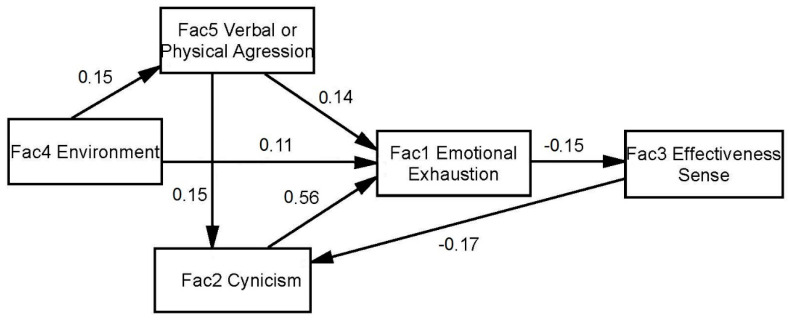
Path Model results. Relationships between the five factors under study converted into exogenous variables to reduce the complexity of the model. Abbreviations: Fac = factor.

Finally, the causality relationships between studied factors were analyzed. To do this, an SEM model (Figure 2) was obtained which includes as observed or exogenous variables those obtained from the sum of the items of each construct.

The obtained causal relationship model provides a clear and logical view of causal dependencies between the factors studied. As “initiators” of the effects on emotional exhaustion there are factors external to the individual: the pressure of the environment and verbal or physical aggression on the referee, doubts as to the usefulness of his work and whether it is worth withstanding such pressures (cynicism) (H2). This state of emotional exhaustion affects your effectiveness in the performance of your activity (H1). Finally, self-perishable inefficiency generates a higher level of cynicism (H3), closing a vicious cycle that causes a permanent decline in efficiency fueled by violence and influence of the environment.

The level of cynicism is the factor that best predicts emotional exhaustion (β = 0.56), they also directly influence the environment (β = 0.11) and indirectly through verbal and physical aggression (β = 0.15 and β = 0.14). It also directly affects verbal and physical aggression on professional efficacy (β = 0.14) and indirectly through cynicism (β = 0.15 and β = 0.56).

In addition, emotional exhaustion directly predicts professional efficacy (β = −0.15), with a negative coefficient, as expected (the higher the exhaustion the lower the efficiency). The rest of the factors influence only indirectly on professional effectiveness. Finally highlight the relationship between professional effectiveness and cynicism (β = −0.17), which closes the ‘vicious circle’ generating a spiral of growth of inefficiency.

The goodness indicators of the model are shown in Table 9, all of them in the good fit interval except the RMR indicator which takes a high value (0.337). This indicator is sensitive to the difference in the range of values taken by each of the variables involved in the model, in our case there are considerable differences (e.g., the range for the cynicism factor is 18 points while for professional efficiency is 11).

## 5. Discussion

The purpose of this study is to examine and empirically prove whether the environment and violence influence burnout in a sample of grassroots football referees. For this, an instrument has been designed composed of five groups of items, three of them associated with the factors Maslach’s questionnaire (emotional exhaustion, cynicism and professional effectiveness), and two groups that represent the psychosocial variables influence of the environment and physical and verbal aggression.

The reliability and validity of each of five groups of items studied has been carried out through an exploratory factorial analysis (EFA) and a confirmatory factorial analysis (CFA). The internal consistencies found in the present study exceed the value of 0.70 recommended by [69], being the emotional exhaustion scale the one with the greatest internal consistency (coefficient ω = 0.91), same result previously obtained by [23] in a sample of Portuguese professional soccer referees.

The results obtained prove the structure of each dimension contained in the hypothetical theoretical model, endorsing the structure of the MBI-GS in three factors according to the original study of the authors [56] and those carried out in our context [62,77]. However, it has been checked that there are some items that do not fully comply with the MBI-GS structure in this collective [78,79].

The present study highlights that the environment the environment can surround grassroots football matches (conflicting players; matches with prominent or influential teams; television broadcasting of the match), as well as the level of verbal and physical aggression to which referees are exposed (disrespect by the public, players or coaches; physical aggression) increase the factors of emotional exhaustion and cynicism, and therefore diminish the sense of effectiveness of their refereeing. These effects are precursors of burnout syndrome in its different phases [17,23].

Previous investigations show that sports referees are among the most stressed people in sports [21,80,81]. Regardless of the league in which they officiate, referees share common aspects based on the functions they perform. The officiating presents high complexity and can become even stressful, not only due to the difficulty of making decisions in a short period of time and keeping order, but also by exposure to criticism and pressure from spectators, players, and coaches [20,21]. The prevalence of stress in the long term could lead to negative psychological and somatic effects such the appearance of work stress and burnout syndrome, which significantly affect job satisfaction, professional commitment and abandonment of their career [17,18,82].

In the sample for this research, it should be noted that referees aged 23 to 24 years (coinciding with the mean age of the cadet and youth categories) are those with the highest values in all factors except in effectiveness sense, thus being the group most susceptible to burnout. In the case of grassroots football referees, the above factors are added the lack of support because in most categories are alone (there are not assistant referees), lack of experience, supports and psychological resources, which can increase the level of burnout. This point is in agreement with findings from earlier studies reporting that younger age and less experience, such as the referees in this sample, are associated with higher levels of burnout [34,83].

By contrast, for the sample’s youngest referees (ages 14 to 18) have low values in verbal or physical aggression and cynicism, means of influence of the environment, and the highest value in effectiveness sense (=29.91), which implies that despite their lack of experience, perceive that their work is correct and important, experiencing a considerable drop (effectiveness sense = 26.72) in the next stretch of age (age 19 to 20 years). This result is at odds with the findings of the investigations cited above. A possible explanation could be related to the type of sample selected, as these are grassroots football referees who are students and very young, while previous research focused on professional league referees. The low levels of burnout in this sample group could be associated by motivation at the beginning in their arbitral career in a non-professional way with high expectations and passion for sport, hoping to achieve some experience to be able to arbitrate to a higher level of league, social relations, travel, fun and some money earned.

Furthermore, the relationships between the different constructs obtained from this study involve confirmation of the three hypotheses initially raised.

On one hand, significant opposite relationships between emotional exhaustion and efficacy (H1) are evident. This finding is in line with those set out above by [41], highlighting as novelty that this is the only direct, channeling cause of the other influences.

On the other hand, it corroborates the existence of direct relationships between the level of physical and verbal aggression suffered by referees and their emotional exhaustion and cynicism (H2). The result achieved would be in line with previous research already cited in the theoretical framework [15,41,42], in which it is evident that fans’ preferences about teams and players, generates a greater number of aggressions and both negatively affect the mood of the referees, generating exhaustion and stress. Reference [46] states that both aggression and cynicism influence emotional exhaustion, an aspect that is strongly evidenced in this study, that presents a strong relationship between cynicism and emotional exhaustion (regression coefficient = 0.56), and a weaker relationship between aggression and emotional exhaustion (regression coefficient = 0.14).

There is also a certain relationship between the influence of the environment and the increase in aggressions, so that the umpires most sensitive to the obstacles provided by the environment also endure a higher level of aggression.

Finally, it is confirmed that self-perception of inefficiency provides demotion, insecurity and doubts about the usefulness of professional activity, i.e., cynicism (H3). The evidence achieved is in line with what was previously exposed by other researchers [23,47,48], who claim that verbal aggression may force a referee to question whether he has made the right decision during the time of the game, which may adversely affect him in terms of his profession, which can lead to dissatisfaction and loss of enthusiasm for his work.

One contribution to highlighting in this study is empirical evidence of the existence of a “vicious circle” provided by the union of the three relationships “emotional exhaustion > professional efficiency > cynicism > emotional exhaustion”, with verbal or physical aggression and the influence of the environment being the initial causes that set in motion and accelerate this loop.

## 6. Conclusions

The environment, especially tense on certain occasions against certain players, spectators or fields perceived as aggressive towards them, is a key aspect. The referee should therefore receive information, training and advice in all those aspects that may improve his performance. These include psychological skills such as concentration, trust, decision making, leadership, styles of interpersonal communication, and emotional control [84].

These psychological competencies require specific preparation, to which few referees devote sufficient attention to today [85,86]. Therefore, it is of great importance, from a practical perspective, the implementation of two intervention proposals: the development of training programs for this group aimed at strengthening personal strategies to deal with obstacles and psychological skills that help reduce the influence of the environment on the preparation of games; and the implementation of education and social awareness programs that reduce violence in the sport, in particular on the collective of referees, including possible sanctions.

Among the limitations of the study, it is worth highlighting the type of incidental or non-probabilistic sample used (composed of volunteer participants, with a 49% response of the population), which makes it difficult to generalize the results. Similarly, some variables have had to be ruled out as it has been determined that the participating referees may not have correctly understood some of the questions, as well as that the cynicism variable is very close to the exhaustion variable.

On the other hand, these results can lead to the advancement of future comparative studies evaluating MBI-GS in environments with different levels of pressure (First Division versus other lower leagues), as well as the need to obtain a sample of a larger group from other places and Committees of Arbitrators, in order to generalize these results to the entire grassroot football referee population.

## Figures and Tables

**Table 1:** Data from the sample of the grassroots football referees.

Experts	N	%
	203	100%
Gender		
Male	194	94%
Female	9	6%
Age (years)		
14–20	66	32.50%
21–25	81	39.80%
26–30	31	15.30%
31–35	14	6.90%
36–40	8	3.90%
>40	3	1.50%
Experience (years)		
0–5	83	40.90%
5–10	93	45.80%
>10	27	13.30%
Categories arbitrated		
Youth	136	67%
Children	131	64.50%
Cadet	126	62%
Juvenile	131	64.50%
Amateur	159	78.30%
Occupation		
Student	160	78.80%
Worked	32	15.80%
Unemployed	11	5.40%

**Table 2:** Structure of the MBI-GS questionnaire adapted to the characteristics of the activity of the participants of the group of grassroot football referees.

Block	Item	Question in Test	Values
**Block1**			
General data	Gender	What is your gender?	Man or Woman
	Age	How old are you?	From 14 to 41
	Experience	What is your experience in arbitration?	From 0 to more than 10
	Categories	In which categories do you referee?	Youth, Children, Cadets, Amateur and Adults
	Occupation	What is your occupation?	Student, Worked, Unemployed
**Block2**			
Environment	Previous 1	Do the teams affect you in your arbitration?	Likert scale: from 0 to 6
	Previous 2	Are you affected by certain known players?	Likert: from 0 to 6
	Previous 3	Are you affected by the recording medium?	Likert: from 0 to 6
**Block3**			
Verbal or physical aggression	Previous 4	Have you ever been disrespected by any coach?	Likert: from 0 to 6
	Previous 5	Have you ever been disrespected by the public?	Likert: from 0 to 6
	Previous 6	Have you ever experienced physical violence?	Likert: from 0 to 6
**Block4 (MBI-GS)**			
Emotional exhaustion	EE1	I am emotionally exhausted.	Likert: from 0 to 6
	EE2	I am “consumed” at the end of a day of arbitration	Likert: from 0 to 6
	EE3	I am tired when I get up in the morning and have to face another day of arbitration	Likert: from 0 to 6
	EE4	All day I am in tension	Likert: from 0 to 6
	EE5	I am “burned” by arbitration	Likert: from 0 to 6
Cynicism	C1	I have lost interest in my job as a grassroot football referee since I started in this position	Likert scale: from 0 to 6
	C2	I have lost enthusiasm for my job as a grassroot football referee	Likert scale: from 0 to 6
	C3	I have become more cynical or ironic about the usefulness of my work as grassroot football referee	Likert scale: from 0 to 6
	C4	I doubt the significance and value of my work as a grassroot football referee	Likert scale: from 0 to 6
Effectiveness Sense	EF1	I can effectively solve all the problems that arise	Likert scale: from 0 to 6
	EF2	I contribute with my actions to the improvement of the arbitration collective	Likert scale: from 0 to 6
	EF3	In my opinion, I am good at my position	Likert scale: from 0 to 6
	EF4	I am encouraged to achieve goals as a grassroot football referee	Likert scale: from 0 to 6
	EF5	I have achieved a lot of valuable things as a soccer referee	Likert scale: from 0 to 6
	EF6	I am sure I am effective as a grassroot football referee	Likert scale: from 0 to 6

Note: EE1, EE2, …, EE5; C1, …, C4; EF1, …, EF6 respectively represent the items of the Emotional Exhaustion factor, Cynicism factor, and Effectiveness Sense factor, of the MBI-GS instrument.

**Table 3:** Descriptive statistics of each factor depending on their gender.

Gender	Statistis	Environment	Verbal or Physical Aggression	Emotional Exhaustion	Cynicism	Effectiveness Sense
Woman (*n* = 9) age * = 19.5	Mean	1.78	9.67	9.22	5.33	27.67
	SD	1.64	3.35	6.94	4.47	2.69
Man (*n* = 194) age * = 24	Mean	1.93	9.79	5.09	3.91	28.40
	SD	2.27	3.48	4.56	4.09	4.97

Abbreviations: *n* = number of referees that are part of that groups; age * = mean age; SD = Standard Deviation.

**Table 4:** Descriptive statistics of each factor depending on their age.

Age Range	Statistic	Environment	Verbal or Physical Aggression	Emotional Exhaustion	Cynicism	Effectiveness Sense
From 14 to 18 *n* = 35	Mean	1.89	9.43	4.74	3.20	29.91
	SD	2.00	3.55	5.43	3.05	3.36
From 19 to 20 *n* = 32	Mean	2.28	8.84	5.47	3.56	26.72
	SD	2.14	3.76	4.55	3.28	5.04
From 21 to 22 *n* = 35	Mean	1.83	9.94	4.66	3.00	28.29
	SD	2.05	2.58	3.16	2.95	4.74
From 23 to 24 *n* = 30	Mean	2.57	11.53	6.27	6.07	28.17
	SD	2.25	3.40	5.43	5.36	4.81
From 25 to 28 *n* = 37	Mean	1.32	8.97	5.38	4.62	28.86
	SD	2.10	3.52	5.76	5.27	5.49
From 29 to 40 *n* = 34	Mean	1.79	10.24	5.26	3.59	28.06
	SD	2.79	3.43	3.72	3.58	5.36
Total sample *n* = 203	Mean	1.92	9.79	5.27	3.97	28.37
	SD	2.24	3.47	4.74	4.11	4.89

Abbreviations: *n* = number of referees that are part of that groups; SD = Standard Deviation.

**Table 5:** Descriptive statistics of each factor according to the category arbitrated.

Category	Statistis	Environment	Verbal or Physical Aggression	Emotional Exhaustion	Cynicism	Effectiveness Sense
Youth (*n* = 136) age * = 22	Mean	1.70	9.65	5.46	4.05	27.90
	SD	2.02	3.46	5.01	4.07	5.12
Children (*n* = 131) age * = 22.5	Mean	1.69	9.87	5.73	4.09	28.19
	SD	2.05	3.42	5.00	4.16	5.14
Cadet (*n* = 126) age * = 23	Mean	1.71	10.03	5.81	4.10	28.20
	SD	2.07	3.32	5.01	4.19	5.13
Juvenile (*n* = 131) age * = 23.5	Mean	1.74	9.85	5.66	3.98	28.15
	SD	2.04	3.36	4.86	4.01	5.06
Amateur (*n* = 159) age * = 24	Mean	2.11	10.28	5.67	4.22	28.62
	SD	2.36	3.35	4.96	4.41	5.08
Total sample (*n* = 203) age * = 23.5	Mean	1.92	9.79	5.27	3.97	28.37
	SD	2.24	3.47	4.74	4.11	4.89

Abbreviations: *n* = number of referees that are part of that groups; age * = mean age; SD = Standard Deviation.

**Table 6:** Correlational analysis of study variables.

	Exhaustion	Cynicism	Efficiency	Environment	Aggression
Exhaustion	-				
Cynicism	0.61 **	-			
Efficiency	−0.24 **	−0.25	-		
Environment	0.21 **	0.13 *	−0.47	-	
Aggression	0.25 **	0.16 *	−0.06 **	0.15	-

Abbreviations: * = *p* < 0.05; ** = *p* < 0.01.

**Table 7:** EFA result on the MBI-GS questionnaire and percentage of variance explained by each factor.

	Factor 1	Factor 2	Factor 3	Factor 4
EE1	0.660	0.471		
EE2	0.724			
EE3	0.776			
EE4	0.787			
EE5	0.578	0.597		
C1		0.802		
C2		0.864		
C3				0.461
C4				0.539
EF1			0.644	
EF2			0.487	
EF3			0.779	
EF4		−0.431	0.411	
EF5			0.395	
EF6			0.598	
Variance	30.84	11.28	7.95	4.09

Note: Extraction Method: Maximum Likelihood. Rotation Method: Varimax with Kaiser Normalization. EE1, EE2, …, EE5; C1, …, C4; EF1, …, EF6 respectively represent the items of the Emotional Exhaustion factor, Cynicism factor, and Effectiveness Sense factor, of the MBI-GS instrument.

**Table 8:** Model adjustment rates associated with the MBI-GS questionnaire.

Fit Index Name	Model Values	Good Fit	Acceptable Fit
Chi-squared (χ^2^)	5.210	0 ≤ χ^2^ ≤ 2df = 74	2df < χ^2^ ≤ 3df = 111
*p*-value	0.052	0.05 < *p* ≤ 1.00	0.01 < *p* ≤ 0.50
df = gr. Freedom	37		
RMSEA	0.045	0 ≤ RMSEA ≤ 0.05	0.05 < RMSEA ≤ 0.08
RMR	0.081	0 ≤ RMR ≤ 0.08	0.08 < RMR ≤ 0.10
NFI	0.949	0.95 ≤ NFI ≤ 1.00	0.90 ≤ NFI < 0.95
CFI	0.984	0.97 ≤ CFI ≤ 1.00	0.95 ≤ CFI < 0.97
GFI	0.952	0.95 ≤ GFI ≤ 1.00	0.90 ≤ GFI < 0.95
AGFI	0.915	0.90 ≤ AGFI ≤ 1.00	0.85 ≤ AGFI < 0.90

Abbreviations: df = degrees of freedom. RMSEA = Root Mean Square Error of Approximation. RMR = Root Mean Square Residual. NFI = Normed Fit Index. CFI = Comparative Fit Index. GFI = Goodness of Fit Index. AGFI = Adjusted Goodness of Fit Index.

**Table 9:** Model adjustment rates associated with the final Path Model.

Fit Index Name	Model Values	Good Fit	Acceptable Fit
Chi-squared (χ^2^)	2.224	0 ≤ χ^2^ ≤ 2df = 6	2df < χ^2^ ≤ 3df = 9
*p*-value	0.527	0.05 < *p* ≤ 1.00	0.01 < *p* ≤ 0.50
df = gr. Freedom	3		
RMSEA	0	0 ≤ RMSEA ≤ 0.05	0.05 < RMSEA ≤ 0.08
RMR	0.337	0 ≤ RMR ≤ 0.08	0.08 < RMR ≤ 0.10
NFI	0.983	0.95 ≤ NFI ≤ 1.00	0.90 ≤ NFI < 0.95
CFI	0.999	0.97 ≤ CFI ≤ 1.00	0.95 ≤ CFI < 0.97
GFI	0.996	0.95 ≤ GFI ≤ 1.00	0.90 ≤ GFI < 0.95
AGFI	0.978	0.90 ≤ AGFI ≤ 1.00	0.85 ≤ AGFI < 0.90

Abbreviations: df = degrees of freedom. RMSEA = Root Mean Square Error of Approximation. RMR = Root Mean Square Residual. NFI = Normed Fit Index. CFI = Comparative Fit Index. GFI = Goodness of Fit Index. AGFI = Adjusted Goodness of Fit Index.

## Data Availability

The data presented in this study are available on request from the corresponding author. The data are not publicly available to protect confidentiality of the research participants.
